# Self-adaptive integration of photothermal and radiative cooling for continuous energy harvesting from the sun and outer space

**DOI:** 10.1073/pnas.2120557119

**Published:** 2022-04-19

**Authors:** Xianze Ao, Bowen Li, Bin Zhao, Mingke Hu, Hui Ren, Honglun Yang, Jie Liu, Jingyu Cao, Junsheng Feng, Yuanjun Yang, Zeming Qi, Liangbin Li, Chongwen Zou, Gang Pei

**Affiliations:** ^a^Department of Thermal Science and Energy Engineering, University of Science and Technology of China, Anhui 230026, China;; ^b^National Synchrotron Radiation Laboratory, University of Science and Technology of China, Anhui 230029, China

**Keywords:** radiative cooling, solar thermal, atmospheric window, self-adaptive spectrum, thermochromism

## Abstract

The sun (∼6,000 K) and outer space (∼3 K) are two significant renewable thermodynamic resources for human beings on Earth. The solar thermal conversion by photothermal (PT) and harvesting the coldness of outer space by radiative cooling (RC) have already attracted tremendous interest. However, most of the PT and RC approaches are static and monofunctional, which can only provide heating or cooling respectively under sunlight or darkness. Herein, a spectrally self-adaptive absorber/emitter (SSA/E) with strong solar absorption and switchable emissivity within the atmospheric window (i.e., 8 to 13 μm) was developed for the dynamic combination of PT and RC, corresponding to continuously efficient energy harvesting from the sun and rejecting energy to the universe. The as-fabricated SSA/E not only can be heated to ∼170 °C above ambient temperature under sunshine but also be cooled to 20 °C below ambient temperature, and thermal modeling captures the high energy harvesting efficiency of the SSA/E, enabling new technological capabilities.

## Significance

The sun (∼6,000 K) and outer space (∼3 K) are two natural energy resources for humans. However, most of the approaches of energy harvesting from the sun and rejecting energy to outer space are achieved independently using absorbers and emitters with static spectral properties. Herein, a spectrally self-adaptive structure with strong solar absorption and switchable emissivity within the atmospheric window (i.e., 8 to 13 μm) is experimentally demonstrated to achieve diurnal solar thermal and nocturnal radiative cooling efficiently. The experiment shows that the proposed structure not only can be heated to 185°C in diurnal mode but also be cooled to −12°C in nocturnal mode. This work opens new possibilities for continuously efficient energy harvesting utilizing the sun and the universe.

Heating and cooling are two kinds of significant end uses of thermal energy in society, which exist in various conditions (e.g., space/water heating, space cooling, and industrial processes) and account for 51% of the total final energy consumption (1). For example, the heating and cooling of buildings are responsible for nearly 48% of the building energy consumption, increasing to be the largest individual energy expense (2). Therefore, heat and cool harvesting relying on clean techniques from renewable energy resources has drawn remarkable attention from fields of engineering to material science because it has considerable potential for global energy conservation and greenhouse emission reduction. Thermodynamically, any heat transportation and work-generation process requires a temperature gradient. The hot sun (∼6,000 K) and cold outer space (∼3 K) are the ultimate heat source and heat sink for the Earth. Theoretical analysis reveals that maximal output work can be extracted from nonreciprocal systems based on the temperature difference between the sun and Earth (∼300 K) with an ultimate solar energy harvesting efficiency limit of 93.3%, while a maximal work of 153.1 W·m^−2^ can also be obtained on the basis of temperature difference between the Earth and outer space (3, 4). Thus, the sun and outer space are two significant renewable thermodynamic resources for the Earth, which can be effectively utilized for clean heat and cool collection.

Photothermal (PT) is a widely used solar thermal collection method that employs solar absorbers to capture solar photons and convert them to heat. Thermal analysis reveals that a good candidate for a solar absorber should have high solar absorptivity and low thermal emissivity simultaneously for efficient solar thermal collection. Various materials, including multilayer metal/ceramic films (5, 6), photonic crystals (7, 8), and metamaterials (9, 10), have been developed for spectrally selective solar absorbers and have been used for real-world applications. Meanwhile, radiative cooling (RC) has re-elicited considerable interest in recent years because it can passively provide clean cooling without any extra energy input (11–14). The waste heat of terrestrial objects can be continuously pumped into the cold outer space, relying on the transparent atmospheric window (i.e., 8 to 13 μm). So, high emissivity within the atmospheric window of materials is necessary for efficient RC, and excellent solar reflection is also important for RC under sunshine. Thus, different materials with the tailored spectrum, such as photonic structures (15–17), structure materials (18), energy-saving paints (19–21), and even metamaterials (22–24), have been reported for passive cooling. On the potential application level, RC implementations also span a range of fields, including passive cooling of buildings (25–27), thermal management of textiles and color surfaces (28–30), atmospheric water harvesting (31), and thermoelectric generation (32, 33). Although the reported PT and RC can generate heat and cold with high efficiency through different spectrally selective materials, most of the approaches are static and monofunctional, which can only provide heating or cooling under sunlight or darkness. Therefore, the dynamical integration of PT and RC for continuously efficient heat and cool harvesting is a new topic for the energy exploitation of the sun and outer space. The tunable combination of PT and RC hybrid utilization has been recently proposed, but mechanical methods such as switching (e.g., flip action) a PT absorber and an RC emitter manually (34) or changing the optical properties of the materials through extra force stimuli (35) are preferred.

Herein, a smart strategy for the dynamic combination of daytime PT and nighttime RC is proposed, corresponding to continuously efficient energy harvesting from the sun and rejecting energy to the universe. A spectrally self-adaptive absorber/emitter (SSA/E) with solar absorption of over 0.8 and emissivity modulation capability of regulating from broadband emissivity of 0.25 within the mid-infrared (MIR) region to the selective high emissivity of 0.75 within the atmospheric window is designed and fabricated for the proof of the concept. Outdoor thermal experimental results demonstrate that the SSA/E can be heated to ∼170 °C above ambient temperature in the daytime PT mode and passively cooled to ∼20 °C below ambient temperature in the nighttime RC mode. Moreover, the heat and cool energy gains of the SSA/E system are respectively predicted to be 78% and 103% larger than those of the reference system that combines static and monofunctional PT absorber and RC emitter.

## Results and Discussion

### Design and Preparation of the SSA/E.

**Fig. 1.**
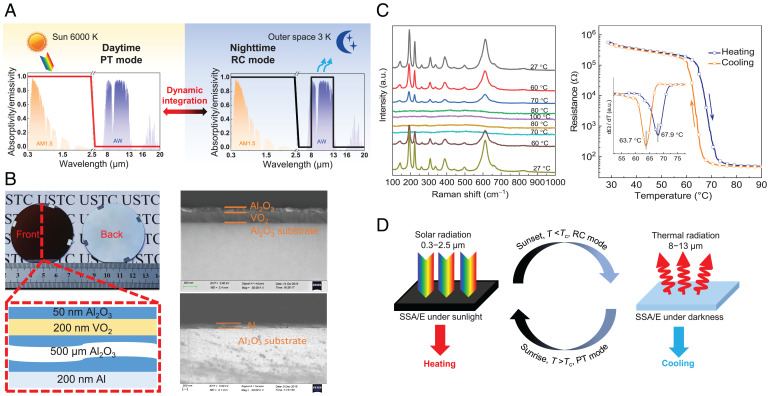
Concept of dynamic integration of PT and RC based on the SSA/E. (*A*) The ideal spectrum requirement of the material for the dynamic integration of PT and RC at day and night. AM1.5: normalized AM1.5 solar spectrum (47); AW: atmospheric window (48). (*B*) Photos and side-view SEM images of the proposed SSA/E with the schematic of the multilayered structure given as reference. A 50-nm-thick Al_2_O_3_ film and 200-nm-thick VO_2_ film are deposited on the front side of a 2-inch-diameter/500-μm-thick Al_2_O_3_ wafer, while the 200-nm-thick metallic Al layer is deposited on the back side. (*C*) The Raman spectra of a 200-nm-thick VO_2_ layer on the Al_2_O_3_ wafer under different temperatures and the resistance measurement during the heating and cooling process. The Raman spectra show phase transition behavior of the VO_2_, and resistance curves reveal the hysteresis behavior of the SSA/E with a critical temperature of 67.9 °C and 63.7 °C for heating and cooling loops, respectively. (*D*) Working principle of the SSA/E for the smart integration of PT and RC. *T* is the temperature of the SSA/E, and *T*_c_ is the critical temperature of the VO_2_ layer. Under sunlight, *T* > *T*_c_, the SSA/E behaves like a PT absorber. Under darkness, *T* < *T*_c_, the SSA/E behaves like an RC emitter.

A concept of dynamic integration of PT and RC is proposed and shown in Fig. 1*A*. During the daytime, the material works like a PT absorber with strong solar absorption and low thermal emissivity in the MIR region. At night, the material emits like a selective RC emitter with unity emissivity within the atmospheric window. Thus, the material can absorb photons through the PT process for solar thermal conversion under sunlight and simultaneously achieve passive cooling at night through the RC process. An SSA/E with a multilayer structure (Fig. 1*B*) is designed and fabricated in accordance with the above-mentioned spectral requirements for the smart integration of PT and RC. A 200-nm-thick vanadium dioxide (VO_2_) film is deposited on a 2-inch-diameter/500-μm-thick aluminum oxide (Al_2_O_3_) wafer, with a 50-nm-thick Al_2_O_3_ film coated on the top surface of the VO_2_ film. In addition, a 200-nm-thick metallic aluminum (Al) film is deposited on the back side of the Al_2_O_3_ wafer. The VO_2_ film is essential for the smart integration of PT and RC because it is optically lossy due to a small bandgap of ∼0.6 eV (36) and it can modulate thermal emissivity because of the intrinsic phase transition behavior (37–39). The thick Al_2_O_3_ wafer not only serves as a substrate for thin-film growth but is also responsible for strong thermal emission due to its selective high emissivity in the atmospheric window (*SI Appendix*, Fig. S1). Moreover, the back Al film is intended to enhance the optical length of the light in the SSA/E and improve its solar absorption, and the thin Al_2_O_3_ thin film acts as an antireflective layer.

Scanning electron microscopy (SEM) and surface morphology testing are conducted to characterize the layered structure of the SSA/E, and the results are shown in Fig. 1*B* and *SI Appendix*, Fig. S2. Besides, the phase transition behavior and quality of the fabricated VO_2_ film are also characterized since this film is essential for the tunable spectral selectivity management of the SSA/E. First, the temperature-dependent Raman spectra testing (Fig. 1*C*, *Left*) shows the existing variations of the sharp Raman peaks during the heating and cooling process, which clearly reflects the reversible metal–insulator transition (MIT) behavior of the VO_2_ film. Second, the hysteresis behavior with a sharp resistance variation of up to four orders of magnitude is observed during the resistance measurement (Fig. 1*C*, *Right*), indicating the high quality of the deposited VO_2_ film on the sapphire wafer. Notably, the differential curves in the inset reveal that the critical temperatures (*T_c_*) of the fabricated VO_2_ film are ∼67.9 °C and 63.7 °C for the heating and cooling loops, respectively, which is remarkably consistent with the reported results of the pure VO_2_ epitaxial film (37). Third, a high-quality, uniform, and epitaxial VO_2_ thin film is also fully evidenced by the resistance distribution (*SI Appendix*, Fig. S3), X-ray diffraction (XRD) characterization (*SI Appendix*, Fig. S4), aberration-corrected scanning transmission electron microscope (STEM) (*SI Appendix*, Fig. S5), and X-ray absorption near-edge spectroscopy (XANES) characterization (*SI Appendix*, Fig. S6).

The working principle of the SSA/E for the smart integration of PT and RC is illustrated in Fig. 1*D*. During the day, the SSA/E will be heated up by solar power due to its solar absorption, and the thermal emissivity of the SSA/E will decrease to a low level when the SSA/E temperature is higher than the critical temperature of the VO_2_ film; this is because the VO_2_ film successfully converts from insulating to metallic phase. Consequently, the SSA/E absorbs like a PT absorber with high solar absorption and low emissivity, which improves the performance of solar thermal collection. At night, the temperature of the SSA/E decreases below the critical temperature of the VO_2_ film due to the absence of sunlight, thus inducing a phase change of the VO_2_ film from metallic to insulating phase, resulting in the emissivity improvement of the SSA/E within the atmospheric window that is the main channel for passive RC. The SSA/E then switches off the low emissive mode and becomes an RC emitter, leading to the passive cooling effect and a further temperature reduction. Overall, the smart integration of PT and RC based on the SSA/E entirely depends on the temperature of the SSA/E, and no extra energy is required for the emissivity switching process.

### Optical Properties of the SSA/E Device.

**Fig. 2.**
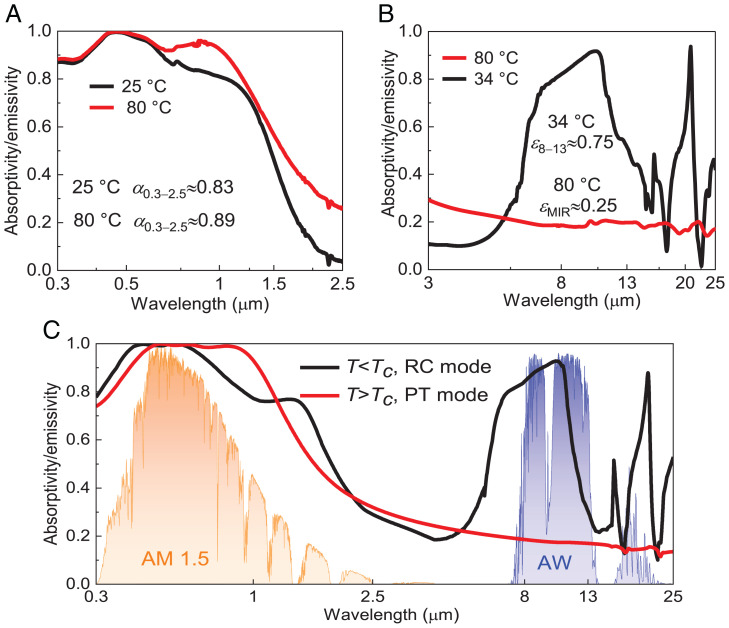
Spectral absorptivity/emissivity of the SSA/E from the UV to the MIR band. (*A*) Measured absorptivity/emissivity of the SSA/E within the solar radiation band under 25 °C and 80 °C. (*B*) Measured absorptivity/emissivity of the SSA/E over the MIR band under 34 °C and 80 °C. (*C*) Simulated absorptivity/emissivity of the SSA/E from the UV to the MIR band when SSA/E temperature is below and above *T*_c_.

The temperature-dependent optical properties of the SSA/E are measured to evaluate its potential for the smart integration of PT and RC. It is found that the fabricated SSA/E exhibits strong absorptivity within the solar radiation band (Fig. 2*A*) due to the intrinsic light absorptivity of the VO_2_ and the antireflection structure of the SSA/E. Specifically, the AM1.5 spectrum weighted solar absorptivity of the SSA/E increases from 0.83 to 0.89 when the temperature of the SSA/E increases from 20 °C (below *T_c_*) to 80 °C (above *T_c_*), corresponding to an enhancement of nearly 7.2%. Importantly, a dramatic regulation effect of the thermal emissivity is observed in Fig. 2*B*. The SSA/E emits like a selective RC emitter with an averaged emissivity of 0.75 within the atmospheric window when the temperature of the SSA/E is below the *T_c_* (i.e., 34 °C; Fig. 2*B*, Inset), which is a good feature for the RC process. However, the SSA/E changes to a surface with low emissivity (i.e., highly reflective) whose average is 0.25 within the MIR region when the temperature of the SSA/E increases above the *T_c_* (i.e., 80 °C; Fig. 2*B*, *Inset*). Theoretically, the predicted spectral absorptivity also proves the strong solar absorption and emissivity modulation property of the SSA/E. Fig. 2*C* shows that the device exhibited substantially high emissions within the atmospheric window when the temperature of the SSA/E is lower than *T*_c_, which was suitable for the nighttime RC mode to reject energy to outer space. When *T* > *T*_c_, the VO_2_ layer converted to the metallic rutile phase, resulting in a large emissivity drop of the SSA/E. Coupled with the solar absorption, the SSA/E can realize efficient PT mode under sunlight.

### Thermal Performance Demonstration of the SSA/E.

**Fig. 3.**
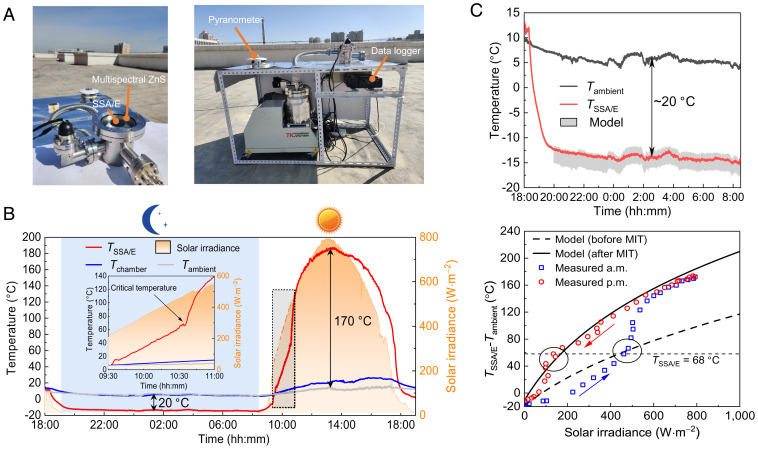
Experimental demonstration of the SSA/E for heating and cooling. (*A*) Photo of the experimental apparatus, which mainly includes a vacuum chamber system and a multispectral ZnS window. (*B*) Twenty-four-hour continuous measurement of the steady-state temperature of the SSA/E in Urumqi. (*Inset*) The MIT transition process of the SSA/E, which indicates the emissivity modulation process. The temperature of the SSA/E (red), the outer surface of the vacuum chamber (blue), the ambient air (gray), and the solar irradiance (yellow, right axis) are presented. (*C*) Comparison of the experimental results with the theoretical predictions during nighttime (*Upper*) and daytime (*Lower*). At night, the gray band is the simulated result considering different parasitic heat loss processes. In the day, the circled area represents the critical temperature zone and two simulation lines correspond to the spectral properties of the SSA/E under PT and RC modes.

An outdoor experiment was conducted on a clear autumn day in Urumqi, China (43′50′′N, 87′35′′E) to explore the thermal performance of the SSA/E under realistic weather conditions. A vacuum chamber (Fig. 3*A* and *SI Appendix*, Fig. S7) coupled with a multispectral ZnS window is applied as the main experimental apparatus, and the SSA/E is fixed in the vacuum chamber with hollow quartz pegs (*SI Appendix*, Fig. S7). The chamber pressure is maintained at the magnitude level of 10^−4^ Pa to maximally suppress the parasitic heat transfer process between the SSA/E and ambient air by convection. The hollow quartz peg support and infrared-reflective aluminum radiation shields (*SI Appendix*, Fig. S8) below the SSA/E are respectively applied to reduce the conduction and radiation heat loss through the backside. Moreover, a multispectral ZnS window with the double-sided antireflection coating is fabricated for the efficient integration of PT and RC modes based on the work of ref. 40, which not only exhibits high transparency in the solar irradiation band and atmospheric window band (*SI Appendix*, Fig. S9) but also provides mechanical support for the high-vacuum environment.

The SSA/E is exposed to the sky from a building roof throughout a 24-h day–night cycle and its stagnation temperature is shown in Fig. 3*B*. The recorded temperature of the vacuum chamber, ambient air, and solar irradiance are provided as references. The relative humidity approximately ranges from 40 to 70% during the night testing period and is within 27 to 49% during daytime testing. The curves show that the temperature of the SSA/E under darkness decreases and reaches an equilibrium state quickly after the SSA/E is exposed to the sky (shortly after 6:20 PM). At night, the temperature of the SSA/E is approximately −12 °C, which is ∼20 °C below the ambient air temperature (Fig. 3 *B* and *C*), indicating that the SSA/E can achieve an efficient subambient cooling phenomenon under the RC mode. When the sun rises (∼10:00 AM), the SSA/E is heated up by the sunlight due to its strong solar absorption, and its temperature rapidly increases. Notably, a sudden change in the SSA/E temperature curve given in Fig. 3*B*, Inset is observed, while the increased rate of solar irradiation is unchanged. This finding indicates that this turning point corresponds to the spectra regulation process of the SSA/E induced by the phase transition of the VO_2_ film. Since the solar absorptivity of the SSA/E is enhanced and the thermal emissivity is suppressed after spectral regulation, the SSA/E can be applied for efficient solar thermal collection. During the diurnal testing, the peak solar irradiation is ∼800 W·m^−2^ over the entire day, and the stagnation temperature of the SSA/E can reach ∼185 °C (∼170 °C above the ambient air temperature), which is much greater than those of demonstrations (80 °C in ref. 41 and 18 °C in ref. 35) for the integration of PT and RC in the previously published papers. At sunset, the solar collection capability of the SSA/E degrades, resulting in a decrease in the device temperature, and then the SSA/E switches to RC mode under darkness.

A theoretical model (*SI Appendix*, Fig. S10 and section 1) for the SSA/E is further developed to predict its thermal performance. The predicted stagnation temperatures of the SSA/E under PT and RC modes both agree well with the measured data (Fig. 3*C*). The theoretically predicted temperatures are mainly determined by the spectral characteristics of the SSA/E, which is closely related to the optical constant variations of the VO_2_ film before and after the MIT transition. The result also shows that two clear turning points around the critical temperature of the VO_2_ film are reflected by the sudden temperature change of the SSA/E (Fig. 3*C*, *Lower*), further confirming that the switching between PT and RC modes is mainly attributed to the phase transition of the VO_2_ film. Accordingly, controlling the *T_c_* value of the VO_2_ film [atomic doping (38)] can further modulate the SSA/E performance within a controllable temperature range, showing considerable potential for energy applications in different situations (winter/summer or cold zone/warm zone). Furthermore, the thermal performance comparison between the SSA/E with the near-perfect spectrum and near-blackbody is performed (*SI Appendix*, Fig. S11). The comparison results show that the proposed tunable and selective spectrum has better PT and RC performance than the blackbody with the static and broadband emissive property.

### Energy Harvesting Potential of the SSA/E Device.

**Fig. 4.**
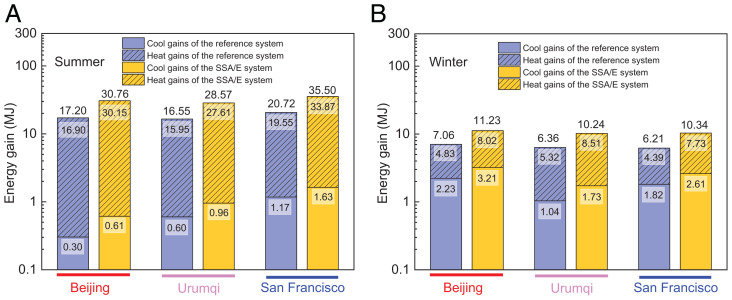
One-day heat and cool energy gains of the SSA/E system in Beijing, Urumqi, and San Francisco. (*A*) in summer and (*B*) in winter. For comparison, a reference system that combines an all-day subambient RC emitter (18) and a selective PT absorber (42) are selected for references.

The proposed SSA/E can absorb solar thermal energy using the PT mode under sunshine and harvest subambient cooling using RC under darkness. Herein, 1-d heat and cool energy gains of the SSA/E system based on the validated theoretical model in summer (Fig. 4*A*) and winter (Fig. 4*B*) of Beijing and Urumqi, China and San Francisco, CA are compared. Moreover, a reference system that combines static and monofunctional subambient RC emitters (18) and selective PT absorbers (42) is selected for comparison. Considering the integration of the SSA/E system and reference systems into the building with the same installation area for heat and cool energy harvesting, the areas of the PT absorber, RC emitter, and SSA/E device are assumed to be 1 × 1, 1 × 1, and 2 × 1 m^2^, respectively. During the simulation, the temperature for solar thermal collection is set as 70 °C and the temperature for RC is set as 5 °C below the local ambient temperature.

Fig. 4 shows that the SSA/E system has the potential to obtain more energy than the reference system. For instance, the heat and cool energy gains of the SSA/E system in summer in Beijing are ∼78% and 103% higher than those of the reference system, respectively, and similar results are also reflected in the summer in Urumqi and San Francisco. For winter conditions, as shown in Fig. 4*B*, the RC capacities of the SSA/E and reference systems substantially increase due to the dry environmental conditions, and the energy gains of the SSA/E system are still much higher than those of the reference system. Taking solar thermal collection as an example, the thermal energy collected by the SSA/E system is 8.02 MJ in Beijing, which is 66% higher than that obtained by the reference system. Even in San Francisco, where the solar irradiance resource is relatively limited, the collected thermal energy of the SSA/E system can still reach 7.73 MJ within 1 d, which is 76% higher than the thermal energy obtained by the reference system (4.39 MJ). The above analysis indicates that the SSA/E system yields relatively better performance than the reference system combining static PT absorber and RC emitter with a single function. Notably, effectively using the collected heat and cool energy is also key for the development of the SSA/E and tunable thermal management strategy. Currently, using an energy storage strategy for renewable techniques has been recognized as a promising method to improve the overall efficiency of renewable energy and reduce the consumption of fossil energy (43), corresponding to greenhouse emission reduction, especially for thermal and cool energy storage due to the developed techniques with cost-effective investment. Thus, the SSA/E can be effectively applied to harvest energy from the hot sun and reject energy to the cold universe by coupling with the energy storage strategy, achieving a continuously efficient and near-passive system for energy harvesting.

## Conclusions

The smart and dynamic integration of daytime PT and nighttime RC is proposed for continuously efficient energy harvesting from the sun and rejecting energy to the universe. An SSA/E with a multiplayer structure of Al_2_O_3_/VO_2_/Al_2_O_3_/Al is optically designed and fabricated for the proof of the concept. The SSA/E strongly absorbs sunlight with an AM1.5 spectra weighted solar absorption of over 0.8 and emits with an emissivity regulation property, which ranges from broadband emissivity of 0.25 within the MIR band to the selective high emissivity of 0.75 within the atmospheric window. The outdoor experimental testing demonstrates that the SSA/E can be heated to ∼170 °C above the ambient temperature in the daytime PT mode and passively cooled to ∼20 °C below the ambient temperature in the nighttime RC mode, thereby showing the switchable macroscopical function of solar heating and subambient RC. Theoretical prediction also shows that the heat and cool energy gains of the SSA/E system are 78% and 103% larger than those of the reference system combining static and monofunctional PT absorber and RC emitter. In summary, this work provides alternative thinking to dynamically explore the thermodynamics potential of the hot sun and the cold universe based on the smart integration of PT and RC, enabling new technological capabilities.

## Materials and Methods

### Design and Preparation of the SSA/E.

The optical properties of the SSA/E are calculated using the transfer matrix method. The reflective indexes of Al and Al_2_O_3_ are obtained from ref. 44. The reflective index of VO_2_ is obtained from ref. 45. During the fabrication, a 200-nm VO_2_ (020) epitaxial film is grown on a 2-inch Al_2_O_3_ (0001) crystal slice with a thickness of 500 μm by radio frequency (rf) plasma-assisted oxide molecular beam epitaxy (OMBE) equipment. During deposition, the substrate temperature is maintained at 550 °C. The metallic vanadium flux is evaporated by an e-beam evaporator, and atomic oxygen flux is generated by an rf-plasma cavity. The film growth rate is calibrated by a quartz-crystal oscillator (thickness monitor) to be ∼0.1 Å/s. Additional details regarding the VO_2_ film preparation by the OMBE technique were previously reported (46). After VO_2_ film deposition, a 50-nm Al_2_O_3_ film is coated on the top surface by rf-magnetron sputtering deposition (LAB 18; Kurt J. Lesker). The rf-sputter power is 250 W, and the growth rate is ∼0.16 Å/s. A 200-nm Al film is then deposited on the back side of the 2-inch sample to form the final SSA/E device.

### Material Characterizations.

The cross-sectional morphologies of the samples are investigated by SEM (Gemini SEM 500; Zeiss). The thickness of the multilayer film is evaluated by a stylus profiler (Dektak XT; Bruker) (*SI Appendix*, Fig. S2). The temperature-dependent electrical measurements are conducted by Keithley 2450 source meters and a customized four-probe system (advanced PW-800) with a variable-temperature sample stage. During the measurements, the temperature sweeping rate is set at 0.1 K/s. The resistance distribution on the 2-inch VO_2_ sample surface is tested at room temperature and 120 °C (*SI Appendix*, Fig. S3). The crystal structure and epitaxial growth of the prepared films are characterized by high-resolution XRD (X′Pert3 MRD, Cu Kα; Malvern) (*SI Appendix*, Fig. S4). The variable-temperature Raman spectra are recorded by an integrated laser Raman system (LabRAM HR; Horiba) with a variable-temperature sample stage. A 532.8-nm He–Ne laser is used as the excitation source. High-angle annular dark-field STEM images were obtained by a JEOL JEM-ARM200F microscope incorporated with a spherical aberration correction system operated at 200 kV with a resolution limit of 0.08 nm (*SI Appendix*, Fig. S5).

### Synchrotron-Based Measurements.

XANES is conducted at the X-ray magnetic circular dichroism (XMCD) beamline (BL12B) in the National Synchrotron Radiation Laboratory, Hefei, China. The total electron yield mode is applied to collect the sample drain current under a vacuum better than 3.75 × 10^−10^ Torr. The energy range is 100 to 1,000 eV with an energy resolution of *ca*. 0.2 eV. The X-ray incident angle is 54.7° (*SI Appendix*, Fig. S6). During the measurement, the sample firmly adheres to the conductive substrate with random orientation, so the polarization dependence is disregarded.

### Optical Characterizations.

The spectroscopic performance of the SSA/E is measured by an ultraviolet-visible near-infrared (UV-VIS-NIR) spectrophotometer (SolidSpec-3700DUV; Shimadzu) and Fourier transform infrared (FTIR) spectrometer (IFS 66v/S; Bruker) for wavelength regions of 0.3 to 2.5 μm and 3 to 25 μm, respectively. The UV-VIS-NIR spectrophotometer is equipped with an absolute specular reflectance attachment. The FTIR spectrometer uses a gold film as a reflectance standard whose reflectivity is taken to be unity across the wavelength region of 3 to 25 μm for reflectivity measurement. A schematic of the variable temperature MIR reflectivity measurement is shown in *SI Appendix*, Fig. S12. The accuracy of the temperature control unit is ±0.5 °C, and the measurement is started when the temperature has been stabilized for 10 min. Then, the spectral absorptivity [*α*(*λ*)] was determined by

[1] α(λ)=1−ρ(λ),

where *ρ*(*λ*) is the spectral reflectivity. Notably, the spectral transmissivity of the SSA/E is zero because a 200-nm-thick Al film is deposited on the back side of the SSA/E.

## Supplementary Material

Supplementary File

## Data Availability

All study data are included in the article and *SI Appendix*.

## References

[1] T. Andre, “Renewables 2020 global status report” (Renewable Energy Policy Network for the 21st Century [REN21], 2020).

[2] US Department of Energy, Heating and cooling. https://www.energy.gov/energysaver/heating-and-cooling. Accessed 8 December 2019.

[3] S. Buddhiraju, P. Santhanam, S. Fan, Thermodynamic limits of energy harvesting from outgoing thermal radiation. Proc. Natl. Acad. Sci. U.S.A. 115, E3609–E3615 (2018). 29610347 10.1073/pnas.1717595115 PMC5910829

[4] W. Li, S. Buddhiraju, S. Fan, Thermodynamic limits for simultaneous energy harvesting from the hot sun and cold outer space. Light Sci. Appl. 9, 68 (2020). 32351692 10.1038/s41377-020-0296-x PMC7181797

[5] Y. Li , Scalable all-ceramic nanofilms as highly efficient and thermally stable selective solar absorbers. Nano Energy 64, 103947 (2019).

[6] A. Al-Rjoub, L. Rebouta, P. Costa, L. G. Vieira, Multi-layer solar selective absorber coatings based on W/WSiAlNx/WSiAlOyNx/SiAlOx for high temperature applications. Sol. Energy Mater. Sol. Cells 186, 300–308 (2018).

[7] K. Cui , Tungsten–carbon nanotube composite photonic crystals as thermally stable spectral‐selective absorbers and emitters for thermophotovoltaics. Adv. Energy Mater. 8, 1801471 (2018).

[8] I. Celanovic, N. Jovanovic, J. Kassakian, Two-dimensional tungsten photonic crystals as selective thermal emitters. Appl. Phys. Lett. 92, 193101 (2008).

[9] Y. Li , Efficient, scalable, and high-temperature selective solar absorbers based on hybrid-strategy plasmonic metamaterials (Solar RRL 8/2018). Sol. RRL 2, 1870196 (2018).

[10] K.-T. Lin, H. Lin, T. Yang, B. Jia, Structured graphene metamaterial selective absorbers for high efficiency and omnidirectional solar thermal energy conversion. Nat. Commun. 11, 1389 (2020). 32170054 10.1038/s41467-020-15116-z PMC7069956

[11] B. Zhao, M. Hu, X. Ao, N. Chen, G. Pei, Radiative cooling: A review of fundamentals, materials, applications, and prospects. Appl. Energy 236, 489–513 (2019).

[12] W. Li, S. Fan, Radiative cooling: Harvesting the coldness of the universe. Opt. Photonics News 30, 32 (2019).

[13] S. Fan, A. Raman, Metamaterials for radiative sky cooling. Natl. Sci. Rev. 5, 132–133 (2018).

[14] D. Zhao , Radiative sky cooling: Fundamental principles, materials, and applications. Appl. Phys. Rev. 6, 021306 (2019).

[15] E. Rephaeli, A. Raman, S. Fan, Ultrabroadband photonic structures to achieve high-performance daytime radiative cooling. Nano Lett. 13, 1457–1461 (2013). 23461597 10.1021/nl4004283

[16] A. P. Raman, M. A. Anoma, L. Zhu, E. Rephaeli, S. Fan, Passive radiative cooling below ambient air temperature under direct sunlight. Nature 515, 540–544 (2014). 25428501 10.1038/nature13883

[17] L. Zhu, A. P. Raman, S. Fan, Radiative cooling of solar absorbers using a visibly transparent photonic crystal thermal blackbody. Proc. Natl. Acad. Sci. U.S.A. 112, 12282–12287 (2015). 26392542 10.1073/pnas.1509453112 PMC4603484

[18] T. Li ., A radiative cooling structural material. Science 364, 760–763 (2019). 31123132 10.1126/science.aau9101

[19] J. Mandal, Y. Yang, N. Yu, A. P. Raman, Paints as a scalable and effective radiative cooling technology for buildings. Joule 4, 1350–1356 (2020).

[20] X. Li , Full daytime sub-ambient radiative cooling in commercial-like paints with high figure of merit. Cell Reports Phys. Sci. 1, 100221 (2020).

[21] X. Li, J. Peoples, P. Yao, X. Ruan, Ultrawhite BaSO_4_ paints and films for remarkable daytime subambient radiative cooling. ACS Appl. Mater. Interfaces 13, 21733–21739 (2021). 33856776 10.1021/acsami.1c02368

[22] Y. Zhai ., Scalable-manufactured randomized glass-polymer hybrid metamaterial for daytime radiative cooling. Science 355, 1062–1066 (2017). 28183998 10.1126/science.aai7899

[23] M. M. Hossain, B. Jia, M. Gu, A metamaterial emitter for highly efficient radiative cooling. Adv. Opt. Mater. 3, 1047–1051 (2015).

[24] X. Zhang, Metamaterials for perpetual cooling at large scales. Science 355, 1023–1024 (2017). 28280168 10.1126/science.aam8566

[25] D. S. Parker, J. R. Sherwin, “Evaluation of the nightcool nocturnal radiation cooling concept: Annual performance assessment in scale test buildings” (US DOE Office of Energy Efficiency and Renewable Energy, 2008).

[26] D. Zhao , Subambient cooling of water: Toward real-world applications of daytime radiative cooling. Joule 3, 111–123 (2019).

[27] E. A. Goldstein, A. P. Raman, S. Fan, Sub-ambient non-evaporative fluid cooling with the sky. Nat. Energy 2, 17143 (2017).

[28] L. Cai , Temperature regulation in colored infrared-transparent polyethylene textiles. Joule 3, 1478–1486 (2019).

[29] P.-C. Hsu ., Radiative human body cooling by nanoporous polyethylene textile. Science 353, 1019–1023 (2016). 27701110 10.1126/science.aaf5471

[30] W. Li, Y. Shi, Z. Chen, S. Fan, Photonic thermal management of coloured objects. Nat. Commun. 9, 4240 (2018). 30315155 10.1038/s41467-018-06535-0 PMC6185958

[31] M. Zhou , Vapor condensation with daytime radiative cooling. Proc. Natl. Acad. Sci. U.S.A. 118, e2019292118 (2021). 33790008 10.1073/pnas.2019292118 PMC8040807

[32] A. P. Raman, W. Li, S. Fan, Generating light from darkness. Joule 3, 2679–2686 (2019).

[33] B. Zhao, G. Pei, A. P. Raman, Modeling and optimization of radiative cooling based thermoelectric generators. Appl. Phys. Lett. 117, 163903 (2020).

[34] J. Liu , Research on the performance of radiative cooling and solar heating coupling module to direct control indoor temperature. Energy Convers. Manage. 205, 112395 (2020).

[35] H. Zhao, Q. Sun, J. Zhou, X. Deng, J. Cui, Switchable cavitation in silicone coatings for energy-saving cooling and heating. Adv. Mater. 32, e2000870 (2020). 32500529 10.1002/adma.202000870

[36] S. Lee , Electronic structure and insulating gap in epitaxial VO_2_ polymorphs. APL Mater. 3, 126109 (2015).

[37] S. Chen , Gate-controlled VO_2_ phase transition for high-performance smart windows. Sci. Adv. 5, eaav6815 (2019). 30931391 10.1126/sciadv.aav6815 PMC6435443

[38] Y. Ke , Adaptive thermochromic windows from active plasmonic elastomers. Joule 3, 858–871 (2019).

[39] M. M. Qazilbash ., Mott transition in VO_2_ revealed by infrared spectroscopy and nano-imaging. Science 318, 1750–1753 (2007). 18079396 10.1126/science.1150124

[40] X. Ao, J. Liu, M. Hu, B. Zhao, G. Pei, A rigid spectral selective cover for integrated solar heating and radiative sky cooling system. Sol. Energy Mater. Sol. Cells 230, 111270 (2021).

[41] Z. Chen, L. Zhu, W. Li, S. Fan, Simultaneously and synergistically harvest energy from the sun and outer space. Joule 3, 101–110 (2019).

[42] L. Zhao , Harnessing heat beyond 200 °C from unconcentrated sunlight with nonevacuated transparent aerogels. ACS Nano 13, 7508–7516 (2019). 31199125 10.1021/acsnano.9b02976

[43] Z. Ding, W. Wu, M. Leung, Advanced/hybrid thermal energy storage technology: Material, cycle, system and perspective. Renew. Sustain. Energy Rev. 145, 111088 (2021).

[44] E. D. Palik, Handbook of Optical Constants of Solids (Academic Press, 1998).

[45] M. A. Kats , Ultra-thin perfect absorber employing a tunable phase change material. Appl. Phys. Lett. 101, 221101 (2012).

[46] L. L. Fan , Growth and phase transition characteristics of pure M-phase VO_2_ epitaxial film prepared by oxide molecular beam epitaxy. Appl. Phys. Lett. 103, 131914 (2013).

[47] National Renewable Energy Laboratory, Reference Solar Spectral Irradiance: ASTM G-173. https://www.nrel.gov/grid/solar-resource/spectra-am1.5.html. Accessed 9 September 2019.

[48] University of Chicago, MODTRAN infrared light in the atmosphere. http://climatemodels.uchicago.edu/modtran/. Accessed 9 September 2019.

