# Supplementary material for: Self-adaptive integration of photothermal and radiative cooling for continuous energy harvesting from the sun and outer space
Source: Proc Natl Acad Sci U S A. 2022 Apr 19;119(17):e2120557119. doi: 10.1073/pnas.2120557119 (PMC9169919; doi:10.1073/pnas.2120557119)
Supplement: Supplementary File [file pnas.2120557119.sapp.pdf]

PNAS
www.pnas.org

Supplementary Information for
Self-adaptive integration of photothermal and radiative cooling for continuous energy harvesting from the sun and outer space

Xianze Ao[1,3], Bowen Li[2,3], Bin Zhao[1,3], Mingke Hu[1], Hui Ren[2], Honglun Yang[1], Jie Liu[1], Jingyu Cao[1], Junsheng Feng[1], Yuanjun Yang[2], Zeming Qi[2], Liangbin Li[2], Chongwen Zou[2,4*], Gang Pei[1,4**]

[1] Department of Thermal Science and Energy Engineering, University of Science and Technology of China, Hefei, Anhui 230026, China.

[2] National Synchrotron Radiation Laboratory, University of Science and Technology of China, Hefei, Anhui 230029, China.

[3] These authors contributed equally to this paper.

[4] Lead Contact

*Correspondence: czou@ustc.edu.cn

**Correspondence: peigang@ustc.edu.cn

This PDF file includes:

Supplementary text
Figures S1 to S12
SI References

Supplementary Information Text

1. Heat transfer model

A theoretical model is developed to predict the stagnation temperature and net output energy flux of the SSA/E. The effect of the pump that maintains the vacuum for testing on the net output energy flux is disregarded during simulation because the vacuum environment can be created at the fabrication process and maintained for a long time when the proposed SSA/E is developed to a commercial product with industrial scales, such as current solar evacuated tubes and high vacuum solar thermal flat plates. Moreover, the clear sky without clouds is selected as the simulation condition. Fig. S10 presents the schematic of the heat transfer of the SSA/E with experimental apparatus. Considering an SSA/E at temperature T_s and a ZnS window at temperature T_w, the heat balance equations of the SSA/E and window are respectively presented as follows:

$$q_{\text{output}}(T_{\text{s}}) = q_{\text{sun,s}} + q_{\text{atm,s}}(T_{\text{amb}}) + q_{\text{w,s}}(T_{\text{w}}) - q_{\text{s,atm}}(T_s) - q_{\text{s,w}}(T_s) - q_{\text{parasitic,s}}(T_s, T_{\text{amb}}), \quad \text{(S1)}$$

$$q_{\text{sun,w}} + q_{\text{atm,w}}(T_{\text{amb}}) + q_{\text{s,w}}(T_s) - q_{\text{w,atm}}(T_{\text{w}}) - q_{\text{w,s}}(T_{\text{w}}) - q_{\text{parasitic,w}}(T_{\text{w}}, T_{\text{amb}}) = 0, \quad \text{(S2)}$$

where the flux item "$q_{\text{a, b}}$" denotes the energy absorbed by b due to emission from a; and subscripts "w," "s," and "atm" represent the ZnS window, SSA/E, and atmosphere, respectively. The flux is defined as

$$q_{\text{sun,i}} = G \cdot \frac{\int_0^\infty F_{\text{sun,i}}(\lambda) I_{\text{AM1.5}}(\lambda) d\lambda}{\int_0^\infty I_{\text{AM1.5}}(\lambda) d\lambda}, \quad \text{(S3)}$$

$$q_{\text{m,n}}(T_{\text{m}}) = 2\pi \int_0^{\pi/2} \int_0^\infty F_{\text{m,n}} I_{\text{BB}}(\lambda, T_{\text{m}}) \sin\theta \cos\theta d\lambda d\theta, \quad \text{(S4)}$$

$$q_{\text{parasitic,s}}(T_{\text{s}}, T_{\text{amb}}) = h_{\text{s}}(T_{\text{s}} - T_{\text{amb}}), \quad \text{(S5)}$$

$$q_{\text{parasitic,w}}(T_{\text{w}}, T_{\text{amb}}) = h_{\text{w}}(T_{\text{w}} - T_{\text{amb}}), \quad \text{(S6)}$$

where i = w or s; G is the solar irradiation; $I_{AM}1.5$ is the AM1.5 spectrum; m, n = w, s, and atm; λ is the wavelength; θ is the zenith angle; $I_{BB}(\lambda,T)=\frac{2hc^2}{\lambda^5[e^{hc/(\lambda kT)}-1]}$ is the blackbody spectral intensity at temperature T, in which h is the Planck's constant, c is the speed of light in the vacuum, and k is the Boltzmann constant; and h_s and h_w are the parasitic heat transfer coefficients of the SSA/E and ZnS window, respectively. The parasitic heat transfer of the ZnS window is due to the convection of the upper surface. The parasitic heat transfer coefficient of the window is $h_w = 2.8 + 3.0u_a$ (1), in which u_a is the wind velocity. For the SSA/E fixed in the vacuum, h_s can be expressed as

$$h_s = h_{air} + h_{rad} + h_{peg}, \tag{S7}$$

where h_{air}, h_{rad}, and h_{peg} respectively correspond to air conduction, radiation, and conduction of the sample stage.

In a vacuum system, the intermolecular interaction is small, and the thermal conductivity of the gas molecule is proportional to the pressure when the mean free path of air molecules exceeds the characteristic length scale of the system. Variable h_{air} is estimated to be 5.2×10^{-4} W·m^{-2}·K^{-1} with a vacuum pressure of 10^{-4} Pa according to kinetic theory (2). Therefore, the parasitic heat loss caused by air conduction and convection is negligible.

The radiative heat loss coefficient is expressed as (2)

$$h_{rad} = \frac{4\varepsilon\sigma T_{avg}^3}{2N}, \tag{S8}$$

where ε is the emissivity of the radiation shields, $\sigma = 5.67 \times 10^{-8}$ W·m^{-2}·K^{-4} is the Stefan–Boltzmann constant, T_{avg} is the average temperature of the vacuum chamber and SSA/E, and N is the number of radiation shields. The absorptivity of the Al radiation

shield is ~0.07 (Fig. S8). During nighttime, the lower bound of the radiative heat loss coefficient $h_{\text{rad-min-n}}$ = 0.03 W·m^{-2}·K^{-1}, with ε = 0.07, T_{avg} = 273 K, and N = 5. In a conservation estimate, N = 1 is assumed, and then the upper bound of the radiative heat loss coefficient $h_{\text{rad-max-n}}$ = 0.16 W·m^{-2}·K^{-1} is obtained. During the daytime, the SSA/E is heated by solar irradiation; thus, T_{avg} = 373 K is assumed, and $h_{\text{rad-min-d}}$ = 0.08 W·m^{-2}·K^{-1} and $h_{\text{rad-max-d}}$ = 0.41 W·m^{-2}·K^{-1} are obtained using the same method.

The conductive heat loss coefficient through the quartz pegs is expressed as

$$h_{\text{peg}} = r\frac{k_{\text{peg}}}{l_{\text{peg}}}, \tag{S9}$$

where r is the ratio between the cross-sectional area of the four quartz pegs and the area of the backside of the SSA/E, k_{peg} is the thermal conductivity of the peg, and l_{peg} is the length of the peg. The outer and inner diameters of the quartz peg are 4 and 2.6 mm, respectively. The diameter of the SSA/E is 50 mm, and then r = 1.48%. With k_{peg}=1.38 W·m^{-1}·K^{-1} (3), and l_{peg} = 0.1 m, we have h_{peg} = 0.2 W·m^{-2}·K^{-1}.

Finally, the parasitic heat transfer coefficient is estimated to be in the range of 0.2−0.4 and 0.3−0.7 W·m^{-2}·K^{-1} in the nighttime and daytime, respectively.

The spectral transfer functions $F_{\text{sun,i}}(\lambda)$ and $F_{\text{m,n}}$ are expressed as

$$F_{\text{s,atm}}(\lambda) = F_{\text{sun,s}}(\lambda) = \frac{\alpha_{\text{s}}(\lambda)\tau_{\text{w}}(\lambda)}{1-\rho_{\text{s}}(\lambda)\rho_{\text{w}}(\lambda)}, \tag{S10}$$

$$F_{\text{w,atm}}(\lambda) = F_{\text{sun,w}}(\lambda) = \alpha_{\text{w}}(\lambda)\frac{1-\rho_{\text{s}}(\lambda)(\rho_{\text{w}}(\lambda)-\tau_{\text{w}}(\lambda))}{1-\rho_{\text{s}}(\lambda)\rho_{\text{w}}(\lambda)}, \tag{S11}$$

$$F_{\text{atm,s}} = F_{\text{atm,s}}(\lambda,\theta) = \varepsilon_{\text{atm}}(\lambda,\theta)F_{\text{s,atm}}(\lambda), \tag{S12}$$

$$F_{\text{s,w}}(\lambda) = F_{\text{w,s}}(\lambda) = \frac{\alpha_{\text{s}}(\lambda)\alpha_{\text{w}}(\lambda)}{1-\rho_{\text{s}}(\lambda)\rho_{\text{w}}(\lambda)}, \tag{S13}$$

$$F_{\mathrm{atm,w}} = F_{\mathrm{atm,w}}(\lambda,\theta) = \varepsilon_{\mathrm{atm}}(\lambda,\theta)F_{\mathrm{w,atm}}(\lambda)\,, \tag{S14}$$

where $\varepsilon_{atm}(\lambda, \theta) = 1\text{-}\tau_{atm}(\lambda)^{1/\cos\theta}$ (4) is the angle-dependent emissivity of the atmosphere, in which θ is the zenith angle, and $\tau_{atm}(\lambda)$ is the atmospheric transmittance in the zenith direction. $\tau_{atm}(\lambda)$ can be obtained using the MODTRAN (5), which considers the effect of air humidity. α, ρ, and τ are the absorptivity, reflectivity, and transmissivity, respectively.

Assuming that the left side of Equation (S1) is set to zero at a steady-state, the two temperatures, namely T_s and T_w, are obtained by solving Equations (S1) and (S2) when the environmental parameters (i.e., solar irradiation, ambient temperature, atmospheric transmissivity, and wind velocity) and spectral characteristics (i.e., absorptivity, reflectivity, and transmissivity) of the SSA/E and window are identified. Moreover, the net energy flux of the emitter can be calculated by solving Equations (S1) and (S2) under the temperature setting of the SSA/E.

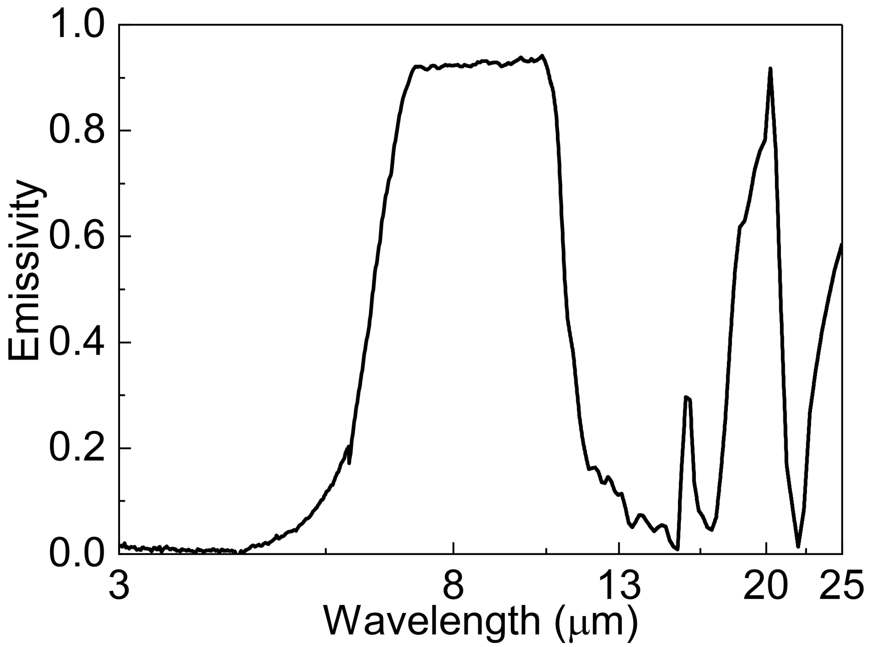

Fig. S1. Measured spectral emissivity of a 500-μm-thick Al_2O_3 substrate within the MIR region.

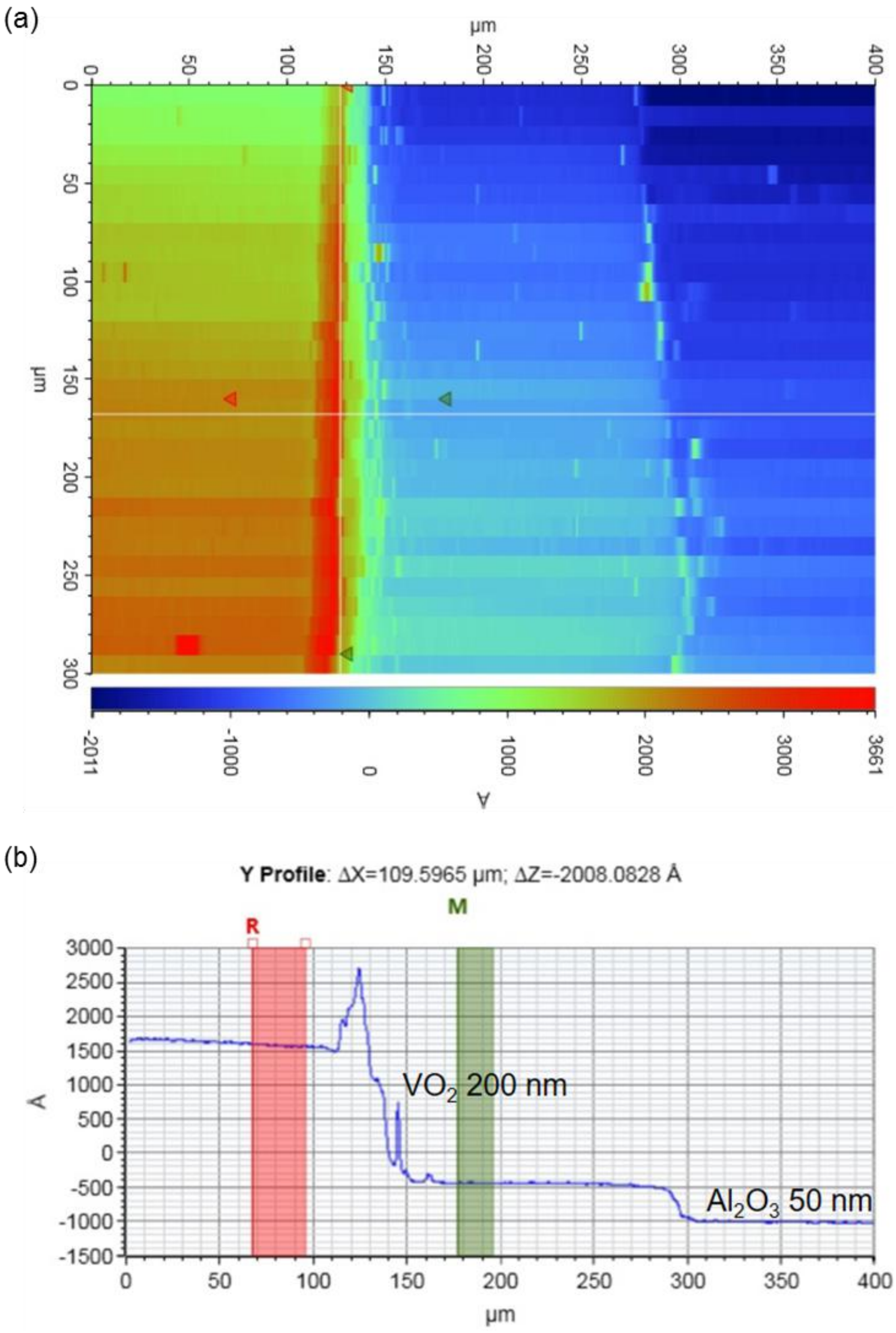

Fig. S2. Surface morphologies for the multi-layer film structure. (a) A 3D surface morphology was produced by the profiler with an area of 400 × 300 um^2. The color bar represents the distribution of height values from low to high. (b) The curve of height values changing with scan length, which shows the specific film thickness of VO_2 and Al_2O_3. The white line shown in the middle of Fig. S2a represents the position of the selected curve.

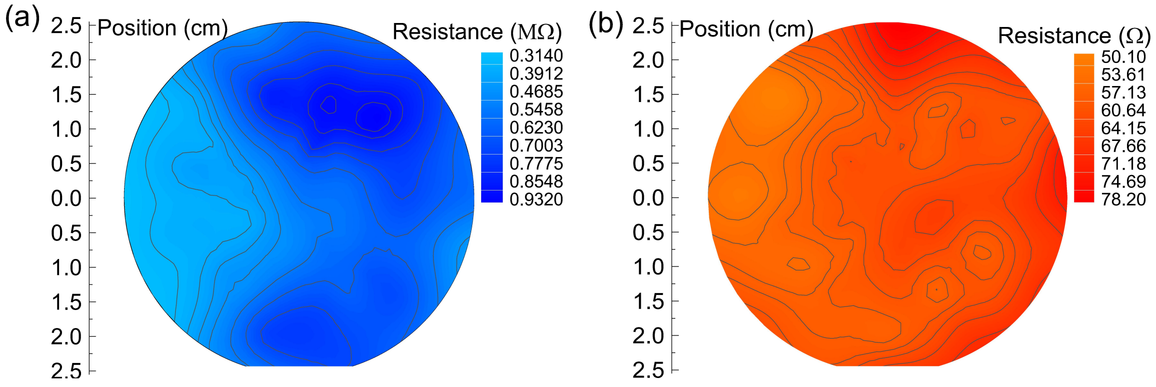

Fig. S3. Electrical measurement for VO_2 layer. Resistance distribution on the two-inch VO_2 sample surface at (a**)** room temperature and (b) 120°C, thereby indicating that the VO_2 thin film has good uniformity.

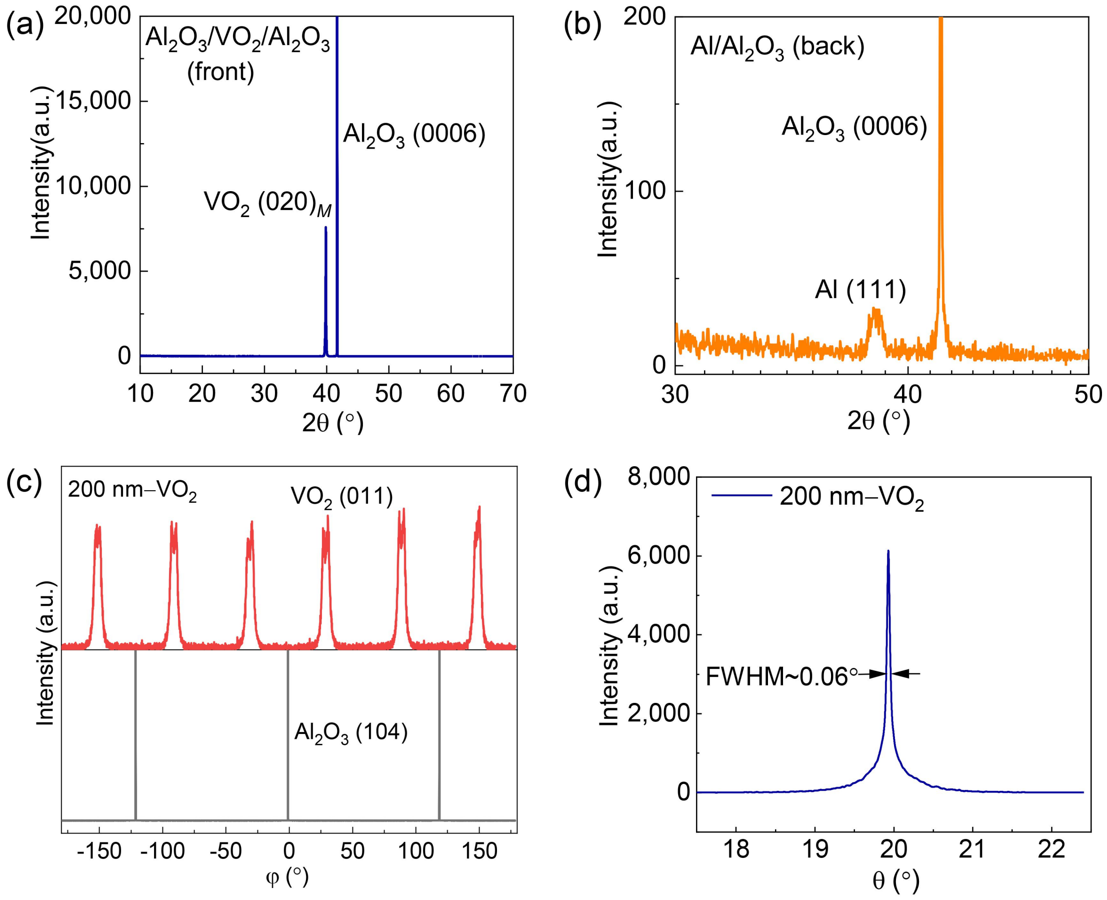

Fig. S4. X-ray diffraction patterns of the SSA/E. θ–2θ scan HRXRD for (a) the two-inch M-VO_2/Al_2O_3 film (front) and (b) the metal Al film (back). The unique diffraction peak of (020) shows the preferred growth orientation. (c) φ-scan mode diffraction shows the epitaxial growth of the VO_2 layer on the Al_2O_3 (0001) surface. (d) Rocking curve of the M-VO_2/Al_2O_3 film with FWHM of ~0.06°, which indicates the perfect crystalline quality of the epitaxial VO_2 film.

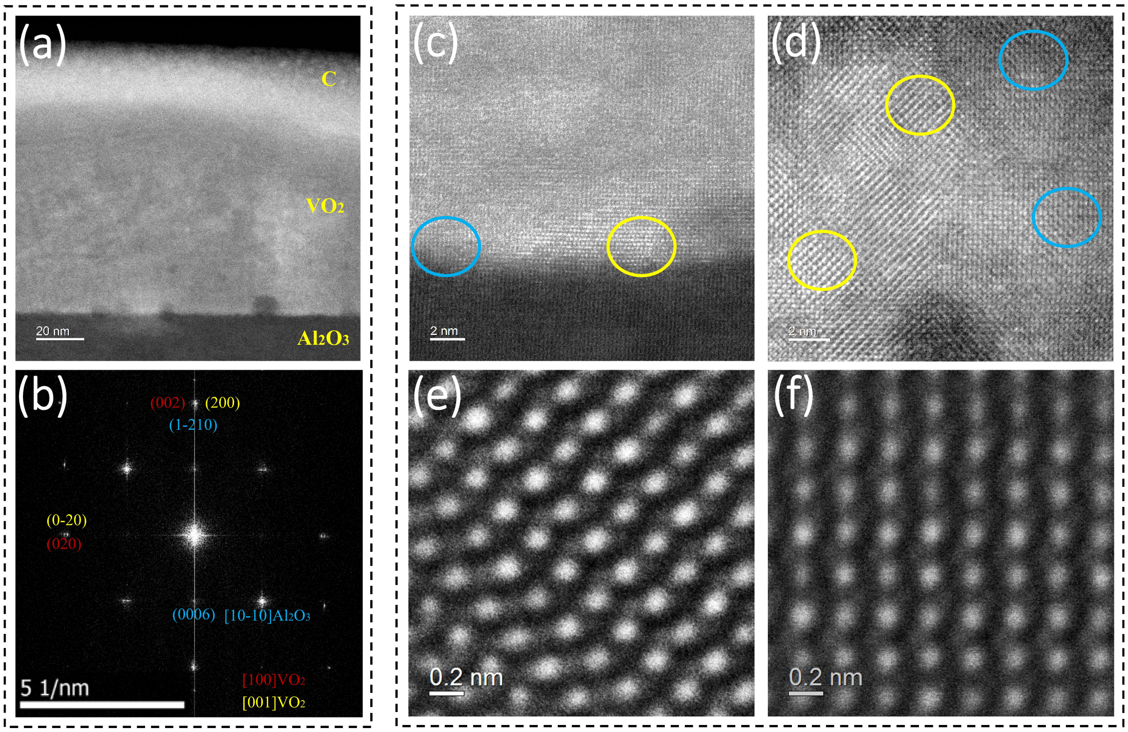

Fig. S5. Scanning transmission electron microscope test for the epitaxial VO_2 film sample. (a) Low-magnification HAADF image taken by STEM showing both orientations growing columnar along the [10-10] sapphire zone axis. (b) Diffraction pattern belong to both crystallites of VO_2 along [001] and [100] and [10-10] substrate. (c) HAADF image taken from the interface between VO_2 and sapphire substrate. The dark region represents the lattice phase of sapphire and two sets of VO_2 domains (marked with blue or yellow circles) can be observed at the interface. (d) HAADF-STEM image shows two sets of VO_2 domains contained within the epitaxial sample. One colored circle indicates the corresponding set of domains. (e) STEM-HAADF high-resolution image of the bright region in (d) (yellow circles) and (f) HAADF image of the dark region in (d) (blue circles).

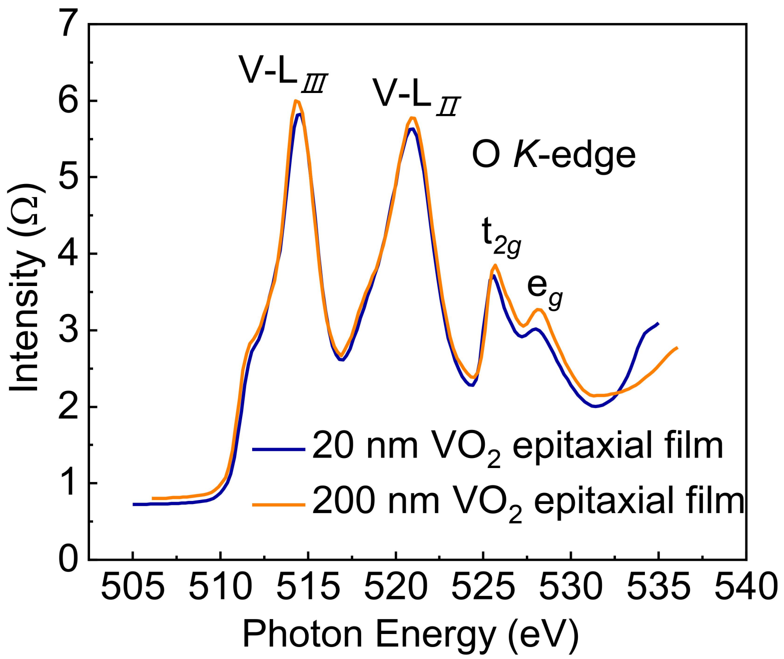

Fig. S6. X-ray absorption near-edge spectroscopy (XANES) measurement. 20-nm-thick and 200-nm-thick epitaxial films have the same absorption peaks, thus indicating the good crystalline quality of the 200 nm VO_2 film.

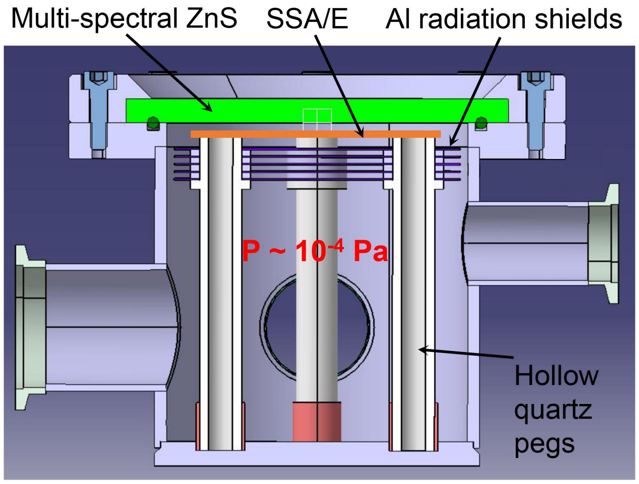

Fig. S7. Schematic of the vacuum chamber.

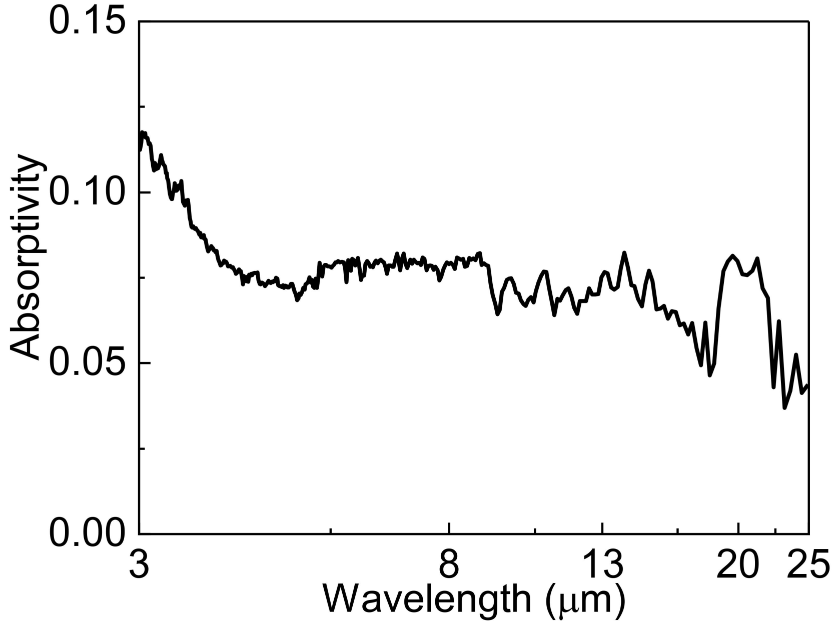

Fig. S8. Optical property of Al radiation shield in the MIR region.

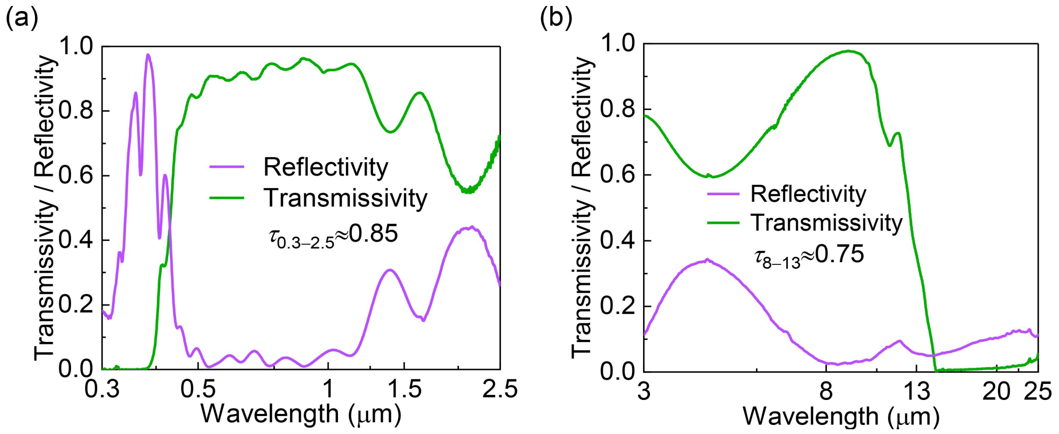

Fig. S9. Measured transmissivity ($\tau(\lambda)$) and reflectivity ($\rho(\lambda)$) of the multispectral ZnS window in the (a) solar and (b) MIR regions.

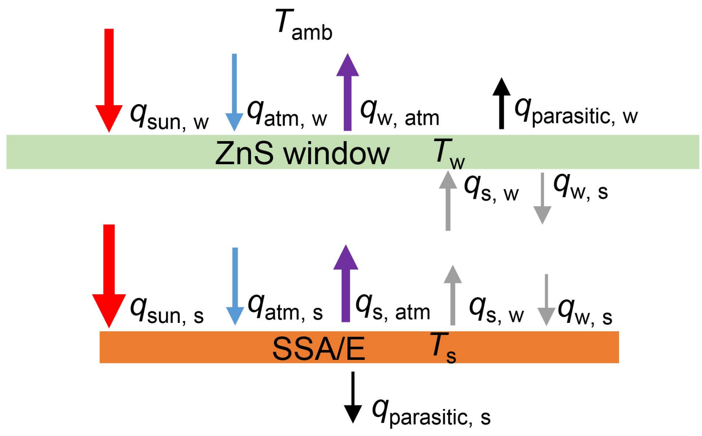

Fig. S10. Schematic of the heat transfer model.

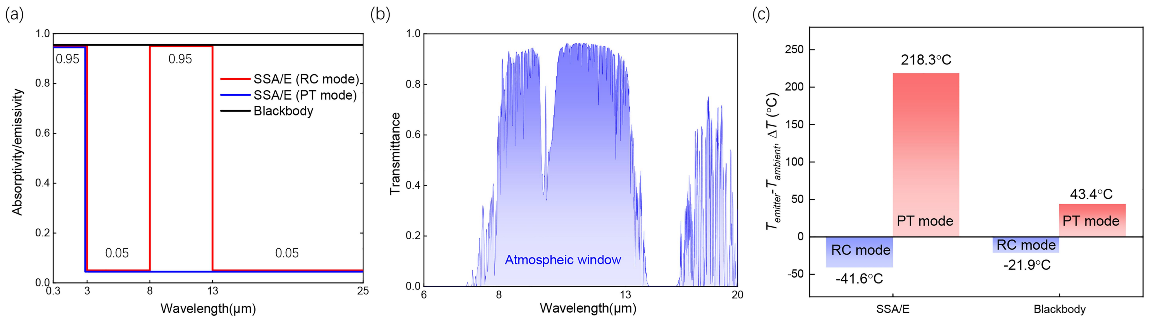

Fig. S11. Thermal performance comparison between the SSA/E and blackbody. (a) The near-perfect spectrum of the SSA/E (PT and RC modes) and blackbody. (b) Atmospheric transmittance used in the calculation. (c) Predicted temperature reductions of the SSA/E and blackbody compared to ambient temperature during nighttime and daytime. Ambient temperature and wind speed are set as 30ºC and 2 m/s, respectively. Solar irradiance is set as 500 W/m^2 at daytime.

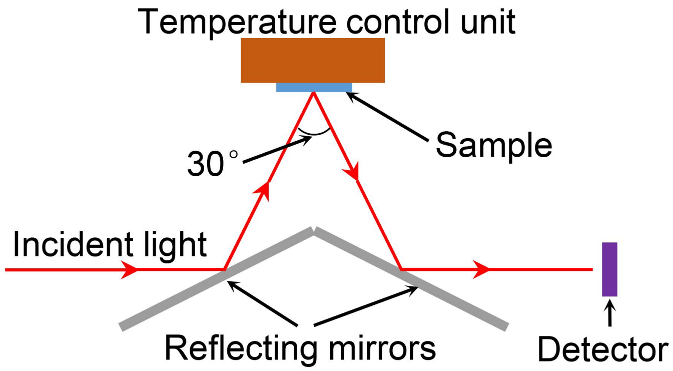

Fig. S12. Schematic of variable temperature reflectivity measurement.

SI References:

1. M. Hu, G. Pei, Q. Wang, J. Li, Y. Wang, J. Ji, Field test and preliminary analysis of a combined diurnal solar heating and nocturnal radiative cooling system. *Appl. Energy* **179**, 899-908 (2016).

2. Z. Chen, L. Zhu, A. Raman, S. Fan, Radiative cooling to deep sub-freezing temperatures through a 24-h day–night cycle. *Nat. Commun.* **7**, 13729 (2016).

3. F. P., Incropera, A. S., Lavine, T. L., Bergman, D. P., DeWitt, *Fundamentals of heat and mass transfer*, Wiley, Hoboken, 2007.

4. A.P. Raman, M.A. Anoma, L. Zhu, E. Rephaeli, S. Fan, Passive radiative cooling below ambient air temperature under direct sunlight. *Nature* **515**, 540-544 (2014).

5. A. Berk *et al*., MODTRAN5: 2006 update. *Proc. SPIE* **6233**, 62331F (2006).
